# Age-dependent flexion relaxation phenomenon in chronic low back pain patients

**DOI:** 10.3389/fbioe.2024.1388229

**Published:** 2024-09-04

**Authors:** Tianwei Zhang, Ali Firouzabadi, Daishui Yang, Sihai Liu, Hendrik Schmidt

**Affiliations:** Julius Wolff Institute, Berlin Institute of Health at Charité - Universitätsmedizin Berlin, Berlin, Germany

**Keywords:** age, low back pain, electromyography, flexion relaxation phenomenon, kinematics

## Abstract

**Background:**

The flexion relaxation phenomenon (FRP) is characterized by suddenly reduced paraspinal muscle activity during full flexion. Previous studies showed significant differences in FRP and flexion angles in chronic low back pain (cLBP) patients compared to individuals without back pain (no-BP). However, the relationship between FRP and flexion angles remains insufficiently understood in older populations. Thus, this study investigated the relationship between FRP and flexion angles concerning to the age and presence of cLBP.

**Methods:**

Forty no-BP subjects (20m/20f; mean age 41.5 years) and thirty-eight cLBP patients (19m/19f; mean age 43.52 years) performed maximum full upper body flexion task. Electromyographic (EMG) measurements were conducted to assess the activity of lumbar erector spinae (ESL), thoracic erector spinae (EST), and multifidus (MF). Lumbar, thoracic, and pelvic angles at the onset (OnsetL/T/P) and offset of the FRP (OffsetL/T/P) and maximum trunk inclination (MaxL/T/P) were calculated. The FRP was evaluated using a flexion relaxation ratio (FRR).

**Results:**

cLBP patients showed smaller FRR in MF and right ESL compared to no-BP individuals (*p* < 0.05), while no differences were found in flexion angles between two groups. Subjects over 40 showed smaller FRR in MF and ESL, and smaller flexion angles on OffsetL and MaxL (*p* < 0.05). Age-related analysis in the cLBP group revealed that patients over 40, compared to younger ones, had smaller FRR in MF and ESL, and smaller values in all thoracic and lumbar flexion angles (*p* < 0.05). While in no-BP group, significant larger flexion angles in OnsetL and OffsetT (*p* < 0.05) were observed in participants over 40. Pain-related analysis in the older group revealed that the cLBP patients, compared to no-BP individuals, had smaller FRR in right MF and right ESL, and smaller values in all lumbar and thoracic flexion angles (*p* < 0.05), while in younger group, there were no significant pain-related differences in FRR, with larger values in all lumbar flexion angles (*p* < 0.05).

**Conclusion:**

Our findings indicate a reduction or absence of FRP in cLBP patients compared to no-BP individuals, with age being a significant factor as those over 40 showed smaller FRP and flexion angles compared to younger individuals.

## 1 Introduction

Low back pain (LBP) is a common musculoskeletal disorder that affects people of all ages and social classes (Airaksinen et al., 2006; Traeger et al., 2021). When the LBP lasts longer than 12 weeks, it is referred to as chronic low back pain (cLBP), with a prevalence of 4.2% in individuals aged 24%–39% and 19.6% in those aged 20–59 (Meucci et al., 2015).

Epidemiologic studies showed that repetitive bending and lifting activities might be linked to an increased risk of developing LBP disorders (Marras et al., 1995; Xu et al., 1997; Swain et al., 2020), possibly caused by accompanying large bending moments in the passive spinal tissues (Adams and Dolan, 1991; Dolan et al., 1994) and large compressive loads due to muscle forces (Schultz et al., 1985; Dolan et al., 1994). During the lumbar full flexion, back muscles are relieved (Floyd and Silver, 1955), and this phenomenon is called the flexion relaxation phenomenon (FRP). FRP is of particular note, as during its occurrence the external moment is carried by the passive spinal tissues (Golding, 1952; Kippers and Parker, 1984; Panjabi, 1992b; a; McGill and Kippers, 1994; Andersson et al., 1996; Du Rose, 2018). The silence of the erector spinae (ES) muscle during FRP is believed to arise from the stimulation of stretch receptors in the posterior disco-ligamentous tissues (Solomonow et al., 2003a; Solomonow et al., 2003b).

Studies comparing FRP between no back pain (no-BP) and cLBP populations reported a greater ratio among the no-BP population (Watson et al., 1997; Colloca and Hinrichs, 2005; McGorry and Lin, 2012; Ringheim et al., 2015; Rose-Dulcina et al., 2020). cLBP patients might reduce their FRP to protect the vulnerable passive structures of the spine, thereby increasing back muscle activity. While this might provide short-term relief, it could increase spinal loads and compromise spinal tissue over time. Such adaptations are considered as potential triggers for LBP (Hodges and Tucker, 2011). Laird et al. (2019) showed that the LBP group had significantly lower lumbar flexion angles and greater lumbar extensor muscle activity than the no-BP group. Dankaerts et al. (2009) also found that LBP patients had significantly lower flexion angles and greater multifidus (MF) activity compared to individuals without back pain.

The influence of age on FRP, however, has been overlooked in the existing literature. While the prevalence of cLBP increases with age, a decrease in lumbar flexion and extension typically starts around age 40 (Sullivan et al., 1994; Galbusera et al., 2014). The spine becomes stiffer as a result of age-related changes, such as reduced water content in the intervertebral discs and surrounding tissues (Solomonow, 2006). In addition, the back muscles of older people do not respond sufficiently to the load on the spine and the activity of the trunk muscles is reduced when performing functional tasks (Hubley-Kozey et al., 2009). The previous study conducted by Kienbacher et al. (2016) compared individuals aged 40–60 with a younger group and found that the older group had significantly lower values in FRP than their younger counterparts. However, it is important to note that the study only focused on cLBP patients.

Thus, the current study aims to investigate the influence of age on FRP for both no-BP and cLBP groups, as well as compare the differences between these groups within matched age ranges. We hypothesized that 1. cLBP patients exhibit significant differences in FRP and flexion angle during trunk flexion compared to no-BP individuals, and 2. Age significantly affects these alterations.

## 2 Methods

### 2.1 Study participants and ethics approval

This observational cross-sectional study was conducted between January 2022 and December 2023 and is part of a 4-year, prospective cross-sectional study to evaluate the influence of various factors on the development and chronicity of LBP. The study was prospectively registered (DRKS-ID: DRKS00027907) and performed in Germany. The Ethics Committee of the Charité–Universitätsmedizin Berlin (registry numbers: EA4/011/10, EA1/162/13) approved this study. All participants were informed about the study’s procedure and signed a consent form. The study included people aged between 18 and 64 years with a body mass index of less than 29. We conducted our study based on previous research that considered 40 years as a threshold for age sub-grouping (Sullivan et al., 1994; Galbusera et al., 2014; Kienbacher et al., 2016).

#### 2.1.1 No-BP group

This group has never experienced pain in the entire back or pelvis and has never had surgery on the spine or lower extremities.

#### 2.1.2 cLBP group

Patients with cLBP (range: >12 weeks to 20 years) and pain intensity based on a Numeric Rating Scale ranging from 0 to 10 (where 0 represents no pain, and 10 is the worst pain imaginable) were included. Patients with prior vertebral fractures, radiculopathies with muscular paresis or previous spinal surgery as well as non-spinal circumstances which diminishes daily activity (respiratory diseases such as COPD, heart failure, myocardial ischemia, neurological disorders, malignancies) were excluded from this study.

### 2.2 Measurement devices and instrumentation

**FIGURE 1:**
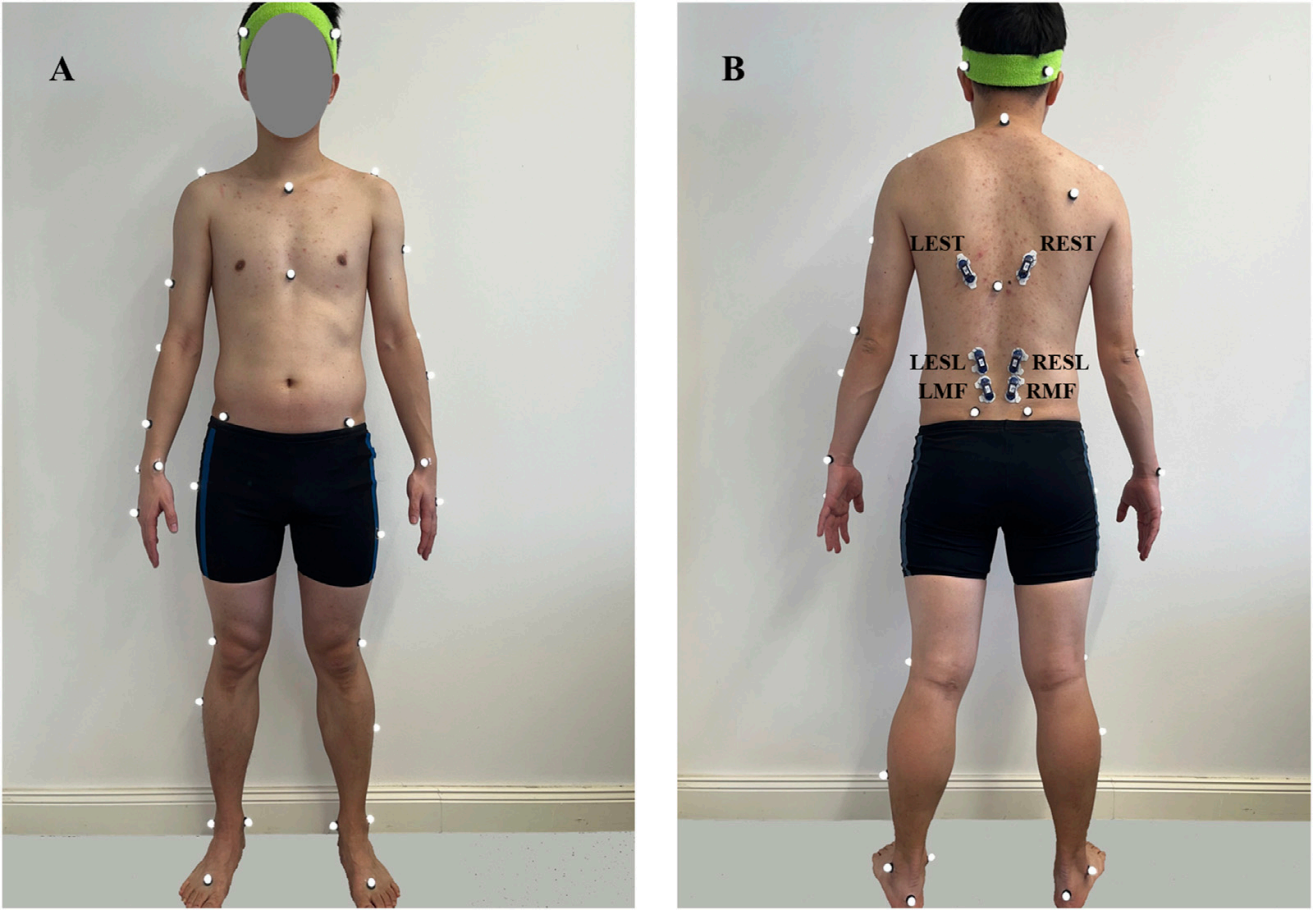
Position of Vicon markers (white ones) and EMG sensors (blue ones) from front **(A)** and back **(B)** views. L/REST: Left/right thoracic erector spinae; L/RESL: Left/right lumbar erector spinae; L/RMF: Left/right multifidus.

The Vicon Motion Capturing System (Vicon Motion Systems, Inc., Oxford, United Kingdom) was used to capture the three-dimensional motion at a sampling frequency of 200 Hz. This included twelve high-speed infrared cameras that track 41 retro-reflective markers (14 mm in diameter) placed on the anatomical landmarks of participants according to the Vicon plug-in gait full-body marker set (Nexus, 2023) (Figure 1).

In addition, a wireless Electromyographic (EMG) system (Myon Aktos, Schwarzenberg, Switzerland) was used to record muscle activities at 1,000 Hz. The skin was shaved, cleaned, and prepped with alcohol before attaching the electrodes. Six surface EMG sensors recorded the muscle activity of the left/right multifidus (L/RMF) (∼2 cm lateral to midline at the L5), left/right lumbar erector spinae (L/RESL) (∼3 cm lateral to midline at the L3), left/right thoracic erector spinae (L/REST) (∼5 cm lateral to midline at the T9) (McGill, 1991; Firouzabadi et al., 2021) (Figure 1). A band-pass filter (30–450 Hz) utilizing a fourth-order Butterworth filter was employed to minimize the noise and artifact effects. A notch filter was used to remove unwanted 50 Hz interference. Following filtration, EMG signals were rectified, and the root-mean-square (RMS) envelopes were then obtained by using a 150-ms moving window and were normalized in relation to their corresponding peak values of maximal voluntary contraction (MVC) (Firouzabadi et al., 2024).

### 2.3 Study protocol

The study protocol consisted of three parts:

1) Study participants were initially asked to complete the following questionnaires:
  - Chronic Pain Grade Questionnaire (CPGQ) to measure the chronic pain severity (Von Korff et al., 1992)
  - Roland-Morris Disability Questionnaire (RMDQ) to assess disability in patients with cLBP (Stroud et al., 2004)
2) Study participants underwent a clinical examination. Here, they were examined by a specialist in orthopedics and trauma surgery with several years of professional experience. Subsequently, the participants were split into a cLBP and a no-BP group.
3) All participants engaged in MVC and flexion exercises. During the MVC test, participants were lying prone on a therapy table, with their upper body extending over the edge and their pelvis and legs fixed on the bed (Konrad, 2006). The experimenter applied resistance to the participants’ shoulders, encouraging them to exert maximum effort.

Participants engaged in pre-trial practice to improve the smoothness of their movements during the task. They were instructed to do the flexion phase with straight knees and standardized upper limb position (arms hanging naturally, relaxed, and perpendicular to the ground) with self-selected velocity, maintain full flexion for 3 s, and then return to the initial position. Each participant performed three trials with a one-minute rest between the trials (Gouteron et al., 2022; Wei et al., 2019; Callaghan and Dunk, 2002).

### 2.4 Data processing

**FIGURE 2:**
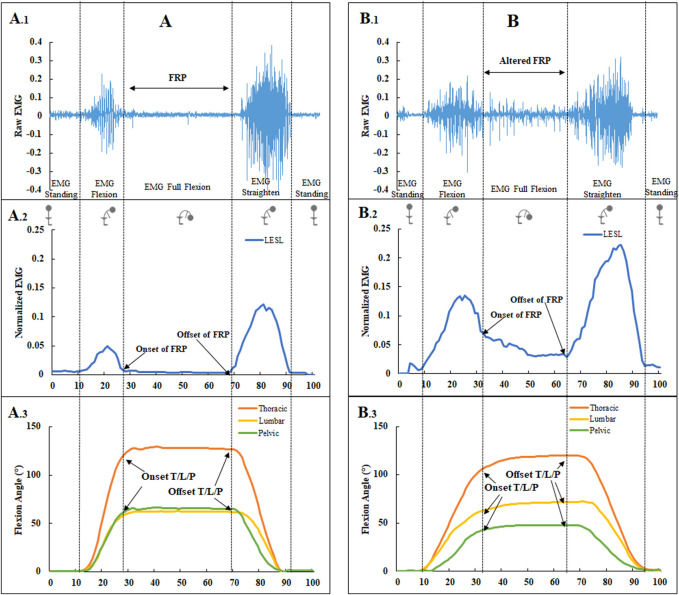
The EMG and flexion angles in a no-BP subject **(A)**. A raw EMG signal from LESL with normal FRP (a myoelectric silence) (A.1). The onset and offset of the FRP from normalized EMG (A.2). The flexion angles of the lumbar, thoracic, and pelvic at the onset and offset of the FRP are based on the percentage of flexion task, progressing from upright standing to full flexion, remaining in full flexion for 3 s, and then returning to upright standing (A.3). The EMG and flexion angles in a cLBP subject **(B)**. A raw EMG signal from LESL with an altered FRP (absence of myoelectric silence) (B.1). The onset and offset of the FRP from normalized EMG (B.2). The flexion angles of the lumbar, thoracic, and pelvic at the onset and offset of the FRP (B.3).

For kinematic analysis, the flexion angles of the lumbar, thoracic, and pelvic at the onset and offset (OnsetT/L/P and OffsetT/L/P) of the FRP as well as at maximum trunk inclination (MaxP/L/T) were calculated (Descarreaux et al., 2008) (Figure 2). The plug-in gait model in Vicon Nexus 2.8.1 was used to identify the relevant frames and calculate segment angles. The lumbar flexion angle was determined by the intersection of the sagittal thoracic and sagittal pelvic axes, with the fixed transverse axis of the pelvis as a reference point. Thoracic angle was defined as the angle formed by the projected sagittal thorax and the sagittal laboratory axes. The pelvic angle was defined as the angle in this plane between the projected sagittal pelvic axis and the sagittal laboratory axis (Nexus, 2023).

MATLAB R2020b (The MathWorks, Inc.) was used to process the EMG data. Two experienced examiners visually determined the standard EMG-off and EMG-on points. The onset of the FRP was indicated by an abrupt decrease in muscle electrical activity during flexion, and the offset of the FRP was indicated by an abrupt increase in muscle electrical activity during extension (Figure 2) (Sarti et al., 2001; Gupta, 2001; Jin et al., 2012; Descarreaux et al., 2008). A flexion relaxation ratio (FRR) for six back muscles was calculated from normalized EMG data by dividing the maximal EMG during the flexion by the maximal EMG during the full flexion as follows (Gouteron et al., 2022):

$$FRR=\frac{\text{Maximum}\;\text{EMG}\;\text{during}\;\text{flexion}\;}{\text{Maximum}\;\text{EMG}\;\text{during}\;\text{full}\;\text{flexion}\;}$$ (1)

### 2.5 Statistical analysis

Statistical analyses were carried out in SPSS version 20 (SPSS Inc., Chicago, United States). Initially, the normality of the data and the homogeneity of variance were confirmed in each group through the utilization of the Shapiro-Wilk test and Levene’s test. For normally distributed data, Analysis of Variance (ANOVA) was employed (*P* < 0.05). Data that did not meet the homogeneity of variances criterion (e.g., MaxT, OffsetP, OffsetT, etc.) were analyzed using the Mann-Whitney U test.

The effect size was calculated based on Cohen’s and Sawilowsky’s recommendation (Cohen, 1992; Sawilowsky, 2009) using G*Power version 3.1.3 (University of Düsseldorf, Düsseldorf, Germany). For our study, with a small effect size of 0.4, an alpha error of 0.05, and a power of 0.8, a minimum sample size of 72 participants was determined to be necessary. Hence, enrolling 78 patients was deemed sufficient to attain the desired statistical power.

## 3 Results

The no-BP group consisted of 40 participants (age: 41.5 ± 13.15 years, BMI: 23.11 ± 2.30 kg/m^2^). The cLBP included 38 participants (age: 43.52 ± 12.65 years, BMI: 23.12 ± 2.23 kg/m^2^). No significant differences were found in demographic characteristics and sex distribution between the cLBP and no-BP groups (*p* > 0.05). However, a significant difference in the RMDQ disability score was found between the cLBP groups younger and older than 40 (*p* = 0.02). Detailed information concerning demographic variables and pain scores can be found in Table 1.

**TABLE 1:** Demographics of study participants. Significant differences (*p* < 0.05) are shown in bold.

	cLBP (n = 38 18 Age<40 20 Age>40)	No-BP (n = 40 20 Age<40 20 Age>40)	*P*-value	Age<40 (n = 38 20 no-BP 18 cLBP)	Age>40 (n = 40 20 no-BP 20 cLBP)	*P*-value
Sex (M/F)	19/19	20/20	1.00	19/19	20/20	1.00
Age (years)	43.52 ± 12.65	41.5 ± 13.15	0.51	31.18 ± 5.27	53.30 ± 7.49	< **0.001**
Body height (cm)	1.76 ± 0.10	1.73 ± 0.11	0.24	1.75 ± 0.10	1.73 ± 0.10	0.32
Body weight (kg)	71.83 ± 11.67	69.84 ± 12.85	0.48	70.37 ± 13.27	71.23 ± 11.35	0.76
BMI (kg/m^2^)	23.12 ± 2.23	23.11 ± 2.30	0.98	22.64 ± 2.61	23.57 ± 1.75	0.07
Pain intensity	3.45 ± 1.9	-	-	2.94 ± 1.77	3.90 ± 1.86	0.14
RMDQ (0–24)	8.00 ± 4.70	-	-	5.47 ± 2.81	10.15 ± 5.00	**0.02**

There were no significant differences in the flexion velocity amongst the sub-groups in our study. The average duration of flexion for individuals with no-BP and cLBP was 2.5 ± 1.2 and 2.7 ± 0.8 s, respectively. For participants under and over 40, the flexion duration was 2.5 ± 0.9 and 2.6 ± 1.1 s, respectively.

Among all of the cLBP patients, twenty (52.63%) showed an altered FRP. The FRR in the cLBP group was statistically smaller than the no-BP group in the LMF (*p* = 0.041), RMF (*p* = 0.019), and RESL (*p* = 0.025), while there were no significant differences for EST and flexion angles for all segments (Table 2).

**TABLE 2:** Separate comparisons of FRR and flexion angles based on pain and age. Significant differences (*p* < 0.05) are shown in bold.

	Pain		*P*-value	Age (years)		*P*-value
	cLBP Mean (SD)	No-BP Mean (SD)		<40 Mean (SD)	>40 Mean (SD)	
FRR						
LMF	4.16 (4.52)	6.15 (3.85)	**0.041**	6.61 (4.23)	3.89 (3.93)	**0.005**
RMF	4.11 (4.66)	6.51 (4.18)	**0.019**	6.92 (4.98)	3.85 (3.56)	**0.002**
LESL	6.85 (6.14)	7.96 (4.69)	0.127	9.34 (5.46)	5.59 (4.81)	**0.002**
RESL	5.81 (4.84)	8.19 (4.29)	**0.025**	9.04 (4.84)	5.13 (3.69)	**<0.001**
LEST	3.18 (2.28)	2.91 (1.88)	0.559	3.55 (2.50)	2.56 (1.45)	0.105
REST	3.68 (2.64)	3.14 (1.73)	0.562	3.88 (2.63)	2.95 (1.66)	0.086
Flexion angle (°)						
OnsetT	104.30 (26.56)	112.01 (23.16)	0.175	111.4 (22.19)	105.18 (27.35)	0.368
OnsetL	51.14 (16.06)	54.70 (12.42)	0.274	55.83 (14.62)	50.24 (13.68)	0.085
OnsetP	53.02 (17.67)	54.31 (14.12)	0.720	54.59 (15.58)	52.82 (16.27)	0.787
OffsetT	113.80 (28.71)	125.02 (14.84)	0.204	123.36 (14.96)	115.94 (28.73)	0.160
OffsetL	56.96 (15.43)	61.97 (10.06)	0.114	62.98 (11.89)	56.26 (13.53)	**0.023**
OffsetP	56.77 (20.85)	61.07 (12.09)	0.313	59.34 (13.57)	58.62 (19.81)	0.853
MaxT	126.65 (17.36)	130.21 (11.94)	0.184	129.5 (13.20)	123.92 (20.34)	0.180
MaxL	59.90 (13.66)	63.61 (8.95)	0.134	64.83 (11.42)	58.92 (11.09)	**0.023**
MaxP	63.61 (17.15)	65.77 (10.72)	0.549	64.48 (12.60)	64.94 (15.67)	0.885

FRR: Flexion relaxation ratio. MaxP/L/T: maximum flexion angle of pelvic, lumbar, and thoracic spine. OnsetT/L/P and OffsetT/L/P: thoracic, lumbar, and pelvic angles at the onset and offset of the FRP.

Participants older than 40 showed statistically smaller values in FRR for the LMF (*p* = 0.005), RMF (*p* = 0.002), LESL (*p* = 0.002), RESL (*p*<0.001), OffsetL (*p* = 0.023) and MaxL (*p* = 0.023) (Table 2).

Age-related analysis in the cLBP group revealed that patients over 40, compared to younger ones, had a statistically smaller FRR in LMF (*p* = 0.017), RMF (*P* < 0.001), LESL (*p* = 0.002) and RESL (*P* < 0.001), and statistically smaller values in all thoracic and lumbar flexion angles (OnsetT (*p*<0.001), OnsetL (*p*<0.001), OffsetT (*P* = 0.005), OffsetL (*p*<0.001), MaxT (*p* = 0.026), and MaxL (*p*<0.001)). While in no-BP group, there were no significant age-related differences in FRR, with statistically larger flexion angles in OnsetL (*p* = 0.033) and OffsetT (*p* = 0.037) (Table 3).

**TABLE 3:** Subgroup analysis based on age by matching the pain. Significant differences (*p* < 0.05) are shown in bold.

	No-BP		*P*-value	cLBP		*P*-value
	<40 Mean (SD)	>40 Mean (SD)		<40 Mean (SD)	>40 Mean (SD)	
FRR						
LMF	7.20 (3.64)	5.10 (3.85)	0.101	5.91 (4.85)	2.67 (3.72)	**0.017**
RMF	7.65 (4.16)	5.37 (3.97)	0.062	6.10 (5.77)	2.33 (2.32)	**<0.001**
LESL	9.08 (5.46)	6.83 (3.55)	0.169	6.83 (3.55)	4.35 (5.62)	**0.002**
RESL	9.27 (4.81)	7.11 (3.49)	0.100	8.78 (4.99)	3.15 (2.73)	**<0.001**
LEST	3.39 (2.29)	3.72 (2.77)	0.138	2.42 (1.23)	2.70 (1.67)	0.130
REST	3.45 (2.14)	2.83 (1.17)	0.376	4.36 (3.08)	3.07 (2.06)	0.073
Flexion angles (°)						
OnsetT	105.64 (26.12)	118.38 (18.26)	0.084	117.98 (14.99)	91.99 (28.91)	**<0.001**
OnsetL	50.52 (14.96)	58.89 (7.49)	**0.033**	61.73 (12.03)	41.60 (13.08)	**<0.001**
OnsetP	53.41 (16.27)	55.22 (11.95)	0.722	55.91 (15.13)	50.41 (19.71)	0.295
OffsetT	119.61 (13.57)	130.43 (14.37)	**0.037**	127.52 (15.70)	101.45 (32.34)	**0.005**
OffsetL	58.96 (11.19)	64.99 (7.96)	0.081	67.44 (11.30)	47.52 (12.32)	**<0.001**
OffsetP	58.94 (11.79)	63.20 (12.30)	0.372	59.79 (15.66)	54.05 (24.71)	0.279
MaxT	128.24 (8.74)	132.18 (14.42)	0.417	130.94 (17.03)	115.65 (22.30)	**0.026**
MaxL	61.10 (9.76)	66.12 (7.46)	0.105	68.98 (11.95)	51.72 (9.35)	**<0.001**
MaxP	66.14 (9.41)	65.40 (12.12)	0.872	62.63 (15.48	64.49 (18.89)	0.693

Pain-related analysis in the older group revealed that the cLBP patients compared to no-BP ones had statistically smaller FRR in RMF (*p* < 0.001), RESL (*p* = 0.003), and statistically smaller values in all lumbar and thoracic flexion angles (OnsetT (*p*<0.001), OnsetL (*p*<0.001), OffsetT (*p* = 0.002), OffsetL (*p*<0.001), MaxT (*P* = 0.014), and MaxL (*p*<0.001)), while in younger group, there were no significant pain-related differences in FRR, with statistically larger values in all lumbar flexion angles (OnsetL (*p* = 0.006), OffsetL (*p* = 0.018), MaxL (*p* = 0.015)) (Table 4).

**TABLE 4:** Subgroup analysis based on pain by matching the age. Significant differences (*p* < 0.05) are shown in bold.

	<40		*P*-value	>40		*P*-value
	No-BP Mean (SD)	cLBP Mean (SD)		No-BP Mean (SD)	cLBP Mean (SD)	
FRR						
LMF	7.20 (3.64)	5.91 (4.85)	0.333	5.10 (3.85)	2.67 (3.72)	0.060
RMF	7.65 (4.16)	6.10 (5.77)	0.136	5.37 (3.97)	2.33 (2.32)	**<0.001**
LESL	9.08 (5.46)	9.63 (5.59)	0.741	6.83 (3.55)	4.35 (5.62)	0.129
RESL	9.27 (4.81)	8.78 (4.99)	0.716	7.11 (3.49)	3.15 (2.73)	**0.003**
LEST	3.39 (2.29)	3.72 (2.77)	0.624	2.42 (1.23)	2.70 (1.67)	0.666
REST	3.45 (2.14)	4.36 (3.08)	0.203	2.83 (1.17)	3.07 (2.06)	0.733
Flexion angle (°)						
OnsetT	105.64 (26.12)	117.98 (14.99)	0.103	118.38 (18.26)	91.9 (28.91)	**<0.001**
OnsetL	50.52 (14.96)	61.73 (12.03)	**0.006**	58.89 (7.49)	41.60 (13.08)	**<0.001**
OnsetP	53.41 (16.27)	55.91 (15.13)	0.633	55.22 (11.95)	50.41 (19.71)	0.346
OffsetT	119.61 (13.57)	127.52 (15.70)	0.066	130.43 (14.37)	101.45 (32.34)	**0.002**
OffsetL	58.96 (11.19)	67.44 (11.30)	**0.018**	64.99 (7.96)	47.52 (12.32)	**<0.001**
OffsetP	58.94 (11.79)	59.79 (15.66)	0.792	63.20 (12.30)	54.05 (24.71)	0.152
MaxT	128.24 (8.74)	130.94 (17.03)	0.520	132.18 (14.42)	115.65 (22.30)	**0.014**
MaxL	61.10 (9.76)	68.98 (11.95)	**0.015**	66.12 (7.46)	51.72 (9.35)	**<0.001**
MaxP	66.14 (9.41)	62.63 (15.48)	0.456	65.40 (12.12)	64.49 (18.89)	0.842

## 4 Discussion

Our study aimed to provide a better understanding of FRP in cLBP patients. We conducted comprehensive evaluations of the FRP and its relationship with trunk kinematics, especially in the context of aging.

### 4.1 FRP and flexion angles manifestation in cLBP patients and No-BP individuals

Consistent with previous studies (Watson et al., 1997; Colloca and Hinrichs, 2005; McGorry and Lin, 2012; Ringheim et al., 2015; Rose-Dulcina et al., 2020), we found that the functional response of FRP was compromised in the cLBP patients. Notably, significantly smaller FRR in the LMF, RMF, and RESL muscles was observed in the cLBP group compared to the no-BP group. This supported the hypothesis that cLBP patients engage in protective behaviors, increasing ES muscle activity to protect the passive structures of the spine (Solomonow et al., 2003a). Interestingly, there were statistically significant differences in both left and right MF, while the ES showed a significant difference only on the right side. This is in line with the findings of Rose-Dulcina et al. (2020), who observed an FRR asymmetry of the ES and hypothesized that the MF may have bilateral adaptability to pain, unlike the ES muscles. In a prior study, patients with unilateral cLBP exhibited bilateral atrophy in the MF at levels L4-5, while the atrophy in the ES corresponded to the side of the pain (Beneck and Kulig, 2012).

The present study further indicates that there were no statistical differences in thoracic FRR between the cLBP and no-BP groups. This observation could potentially be attributed to the focal point of pain experienced by our cLBP patients, primarily localized in the lumbar region rather than the thoracic region. Furthermore, in the no-BP group, the thoracic FRR was smaller compared to the lumbar FRR. Considering the FRR calculation formula (Equation 1), the smaller thoracic FRR suggests that the thoracic muscles remain activated during full flexion, in contrast to the reduction of activity (larger FRR value) observed in the lumbar muscles. This finding was supported in a study by McGill and Kippers (1994), where they showed that most of their no back pain participants could completely relax their lumbar extensors during the full flexion phase, while their thoracic extensors remained active. Furthermore, the study conducted by Callaghan and Dunk (2002) showed that EST is more likely to exhibit FRP during full flexion in a sitting position.

Although cLBP patients had smaller lumbar flexion angles as compared to no-BP individuals, the difference was not statistically significant. This suggests that while flexion angles might be affected in cLBP patients, it may not be the sole or most potent factor delineating them from no-BP individuals. Consistent with the findings of other studies (McGorry and Lin, 2012; Sánchez-Zuriaga et al., 2015; Firouzabadi et al., 2024), there were no significant differences in trunk flexion angles between the cLBP and no-BP groups. However, there was notably larger ESL activity in cLBP participants during full flexion task, even though these cLBP patients were assessed during pain-free intervals (Sánchez-Zuriaga et al., 2015). It suggests that relying solely on maximum flexion angle measurements may not adequately capture the distinctions in flexion-extension movements between those with cLBP and their no-BP counterparts.

### 4.2 Age as a significant modifier

The analysis of the FRP across different age groups revealed that individuals over 40 demonstrated a statistically reduced FRR in LMF, RMF, LESL, RESL, and limited flexion angles in OffsetL and MaxL. These findings were supported by previous studies that showed heightened back extensor muscle activity (Quirk and Hubley-Kozey, 2014; Kienbacher et al., 2015) and statistically lower FRP in older adults compared to their younger counterparts (Kienbacher et al., 2015). Moreover, our results were also consistent with previous research that has linked a decrease in the flexion angles for lumbar flexion with aging, commencing typically around the age of 40 (Sullivan et al., 1994; Galbusera et al., 2014). Reduced water content in intervertebral discs and the loss of viscoelastic properties in posterior ligaments (Solomonow, 2006; Hubley-Kozey et al., 2009) might be the causes of these alterations. This suggests that older adults are more inclined with increased extensor muscle activity to compensate for the reduction in lumbar mobility compared to younger individuals.

Age-related subgroup analysis in the cLBP group revealed that the patients over 40, compared to younger ones, showed statistically smaller FRR in bilateral MF and ESL and smaller values in all lumbar and thoracic flexion angles. The significantly higher disability score (RMDQ-24, Table 1) in cLBP over 40 could explain it, as a previous study found that the participants with higher RMDQ scores demonstrated lower FRP levels and higher lumbar spine global stiffness (Xia et al., 2017). Moreover, our results are also consistent with the study conducted by Kienbacher et al. on 216 patients; significant differences in the ratio at half and maximum trunk flexion were found among age groups, with the largest values in the youngest group and the smallest in the oldest group; furthermore, age influenced task-specific lumbothoracic changes in angles, wherein the oldest group showed the lowest values and the youngest group displayed the highest values (Kienbacher et al., 2016).

Although previous studies have investigated the FRP and flexion angles in cLBP patients, our study further compared the FRP and flexion angles in both no-BP and cLBP groups within matched age ranges. Pain-related analysis within the older group revealed that the cLBP patients, compared to no-BP individuals, showed statistically smaller FRR for RMF and RESL and smaller values in all flexion angles for lumbar and thoracic. However, within the under-40 age group, our findings showed that the lumbar flexion angles in patients with cLBP are significantly larger than in no-BP individuals. Previous research (Solomonow, 2006; Hubley-Kozey et al., 2009) showed that the degenerative changes in the intervertebral disc and adjacent structures result in subtle alterations in the mechanical properties of the functional spinal unit. Loss of viscoelastic mechanical properties and degeneration of spinal discs and adjacent structures have been repeatedly associated with aging. A tendency toward spinal stiffening as degeneration increases has been observed in some studies (Kettler et al., 2011; Galbusera et al., 2014). This stiffness can potentially disrupt the regular input of the ligaments, subsequently contributing to proprioceptive deficits, and these deficits may lead to alterations in muscle recruitment patterns (van Dieën et al., 2003; Keenan et al., 2005; Georgy, 2011). Therefore, the changes in lumbar flexion angles in individuals over 40 might be more closely associated with physiological alterations in the patient rather than the reduction in flexion angles caused by pain. The decreased lumbar mobility may cause older individuals with cLBP to activate more muscles to maintain stability during the entire flexion task, resulting in a lower FRR in those over 40 with back pain.

While our study presented significant findings, certain limitations must be acknowledged. Firstly, the cLBP cohort exhibited relatively mild to moderate pain intensity, and we did not investigate the effect of pain intensity on FRP. However, Alschuler et al. (2009) demonstrated an association between pain intensity and FRP in LBP patients. Additionally, factors such as the duration of cLBP and psychological and occupation parameters, which might influence FRP and flexion angles, were not thoroughly evaluated. Furthermore, due to the small sample size of participants over 55 years old (7 cLBP, eight no-BP) and under 30 years old (7 cLBP, 10 no-BP) in our study, we did not further perform subgroup analysis on participants over and under 40. Future studies with more extensive, diverse cohorts and multi-dimensional evaluations could provide more nuanced insights.

## 5 Conclusion

The present study showed a nuanced relationship between the flexion-relaxation phenomenon, lumbar flexion angles, and age, especially in cLBP; FRP is reduced in cLBP patients, and age significantly alters FRP, especially in old cLBP patients. The findings emphasize the need for comprehensive evaluations and tailored therapeutic interventions to manage cLBP, factoring in biomechanical and age-related changes.

## Data Availability

The raw data supporting the conclusions of this article will be made available by the authors, without undue reservation.

## Abbreviations

LBP, Low back pain; cLBP, Chronic low back pain; No-BP, No back pain; RMS, Root mean square; EMG, Electromyography; MVC, Maximum voluntary contraction; FRP, Flexion relaxation phenomenon; FRR, Flexion Relaxation Ratio; MF, Musculus Multifidus; ES, Musculus Erector spinae; L/RMF, Left/right multifidus; L/RESL, Left/right lumbar erector spinae; L/REST, Left/right thoracic erector spinae; OnsetL/T/P and OffsetT/L/P, Thoracic, lumbar, and pelvic angles at the onset and offset of the FRP; MaxP/L/T, The maximum range of motion of the pelvic, lumbar, and thoracic parts during flexion.
